# Early Operation in Strangulated Hernia*Read before the Medical Association of Georgia, April, 1894.

**Published:** 1894-07

**Authors:** William Perrin Nicolson

**Affiliations:** Atlanta, Ga., Prof. of Anatomy in Southern Medical College


### EARLY OPERATION IN STRANGULATED HERNIA.*

By WILLIAM PERRIN NICOLSON, M.D., ATLANTA, GA.,
Prof. of Anatomy in Southern Medical College.

There is probably no surgical affection in which the result of operative interference depends so much upon prompt action as in strangulated hernia, and the fact that almost every case first falls into the hands of the family physician, who is a general practitioner, leads me to offer a plea for early operation in cases where the fact of strangulation is well established, and to urge that should he himself be unwilling to operate, no time should be lost in calling in the assistance of some one who can.

Statistics show beyond any doubt that where a fatal issue follows this operation, it may, in almost every instance, be traced to either the pathological change that has taken place in the intestine, or the exhaustion of the strength of the patient by prolonged pain and vomiting. The modern surgical methods have perfected the operation, so that it has been removed from the category of capital, and transferred to that of minor, operations, provided it is done before the above mentioned conditions have arisen.

The time in which destructive changes may take place in an intestine completely strangulated varies, but the fact that they may

*Read before the Medical Association of Georgia, April, 1894.

occur in a few hours should always be borne in mind in deciding upon operation. In a case under my own charge where strangulation suddenly occurred, though the operation was done within a little less than four hours after full development of symptoms, the intestine when exposed was found perfectly black, and would have undoubtedly been necrosed in a few more hours. In this instance delay would certainly have resulted in a far more dangerous operation, and, in all probability, in the death of the patient.

The question may be asked, what time should be selected to operate? and, as a general proposition, it may be laid down as a safe rule that, when taxis fairly tried under an anesthetic fails to reduce the hernia, the time for operating has arrived. I am well aware that many advocate the resort to hot baths, ice applications, and other means directed to relaxing the constrictions and reducing the congestion, but there are in my opinion few arguments to bear out this line of treatment. The constriction as a rule is one that does not relax, on account of the nature of the tissues which compose it, and the congestion is of a passive nature, due to the fact that the arterial blood entering is not obstructed, while the return current through the veins is. If these steps are to be taken, I should rather advise their use before resorting to an anesthetic, and when circumstances demand the administration of ether as a final step, the surgeon should be prepared to operate before the patient regains consciousness from the anesthetic. Continued efforts at taxis after this can only serve to further imperil the life of the intestine, and place it in a condition that will give rise in the operation to the serious question of its viability if returned into the abdomen, and when it is reduced its nerve force and circulation may be so impaired that intestinal obstruction will remain even after the bowel has been returned into the abdomen. Another serious result of continued taxis may be that a hernia finally reduced into the abdomen may be in a condition that, had the operator enjoyed the privilege of inspecting it, he would not have returned it at all.

In addition to this, as evidenced by things under my own observation, a hernia that has been strangulated for a number of days, may finally pass back into the abdomen and be in such a condition that it is unable to resist pressure, and a rupture will take place, permitting extravasation of its contents into the abdominal cavity. In this connection it may be stated that in this day no good surgeon will permit the intestine to return into the abdomen by operation, without opening the sac and inspecting it. This was formerly advised as the proper step before the days of clean surgery, during which period may also be observed the fact that in continued obstruction of the bowels after return of the intestine a subsequent rupture was frequently observed.

Lastly, I would state that my only purpose in presenting this subject is the hope of impressing upon those into whose hands such cases fall, that if reduction cannot be early accomplished, and they are themselves unwilling to operate, they should give the patient the benefit of the services of one who will, before the condition shall have been converted from one of comparatively a simple nature, to one of the gravest conditions that exist in surgery. In conclusion I would offer as the reasons for thus urging early operation the following:

*First.* That the operation *per se* is practically devoid of danger.

*Second.* The dangers that attend upon operation are due to results of delay; namely, the changes in the intestine and exhaustion of patient.

*Third.* That as a general proposition where strangulation has existed for any length of time, it is safer to operate, and then have the opportunity to inspect the bowels before returning, even than to reduce it by simple taxis.

*Fourth.* A strangulated intestine may finally return by taxis only to remain in a state of paralytic obstruction, or to finally rupture into the peritoneal cavity.

*Fifth.* When early operation is permitted, and the patient's strength has not been exhausted, it can easily be converted at the same time into an operation for the radical cure of the hernia, and thus relieve the patient from after dangers that may arise.

